# Clinical Outcomes of Pharmacist Involvement in Cardiac Arrest and Trauma Resuscitations: A Scoping Review

**DOI:** 10.3390/pharmacy13040089

**Published:** 2025-06-24

**Authors:** Harshita Patel, Myles Wee, Aaron M. Tejani, Anthony Lau

**Affiliations:** 1 Faculty of Pharmaceutical Sciences, University of British Columbia, Vancouver, BC V6T 1Z3, Canada; hpatel16@student.ubc.ca (H.P.); myleswee@gmail.com (M.W.); 2 Lower Mainland Pharmacy Services, Fraser Health Authority, Vancouver, BC V2Y 0A1, Canada; aaron.tejani@fraserhealth.ca; 3 Emergency Department, Vancouver General Hospital, Vancouver, BC V5Z 1M9, Canada

**Keywords:** advanced cardiac life support, cardiac arrest, emergency department, pharmacists, trauma resuscitations

## Abstract

Background: The role of clinical pharmacists in the emergency department continues to gain recognition, particularly during cardiac and trauma resuscitations. However, their contributions to patient outcomes remain unclear. The objective of this scoping review with narrative synthesis was to determine the impact of pharmacists on medication and patient outcomes during cardiac and trauma resuscitations and to identify barriers to integration. Methods: A literature search of databases in September 2024 identified randomized and non-randomized control trials, evaluating the impact of pharmacists’ involvement in cardiac or trauma resuscitations. Excluded were studies on acute stroke, acute hemorrhage, and sepsis. Data were extracted and analyzed for primary (e.g., medication errors and Advanced Cardiovascular Life Support [ACLS] compliance) and secondary outcomes (e.g., pharmacists’ education and training). Results: Of the 560 records screened, 26 records were included in the final analysis. Due to heterogeneity, quantitative analysis was not feasible. Among primary outcomes, ACLS guideline compliance and medication errors were commonly reported; mortality and length of stay were less commonly reported. ACLS certification improved pharmacists’ confidence in their tasks. Pharmacists’ presence also correlated with reduced healthcare costs. Conclusions: Our analysis suggests that the involvement of pharmacists in the context of emergency cardiac or trauma resuscitations may benefit direct patient outcomes and indirect outcomes.

## 1. Introduction

Over the past decade, clinical pharmacists have increasingly been recognized as integral members of cardiac arrest and emergency trauma teams in North America and globally. This recognition is driven by growing evidence of their clinical impact, organizational endorsement (e.g., by the American College of Emergency Physicians), and increased integration into Advanced Cardiovascular Life Support (ACLS)-certified resuscitation teams [1,2,3,4,5,6]. Clinical pharmacists are now routinely included in emergency response teams in many hospitals, where they contribute to real-time decision-making and medication management during high-acuity events. The current literature suggests that pharmacist involvement in emergency response teams leads to a reduction in medication errors, improved adherence to ACLS protocols, decreased time to medication administration, and potential cost savings for institutions [7,8]. Their key roles include the preparation and administration of medications, provision of drug information, therapeutic recommendations, and documentation support [7,8,9]. Several observational studies have demonstrated that pharmacists can help reduce time to analgesia and other time-critical therapies during resuscitations, reduce medication errors, improve protocol compliance, and support closed-loop communication within multidisciplinary teams [10,11,12]. However, the impact of pharmacists’ involvement on medication effectiveness and clinical outcomes (e.g., length of stay, morbidity, and mortality) in cardiac resuscitation teams remains unclear [13,14,15]. By clarifying the extent of these services and which areas pharmacists can contribute to the most, a clearer and guiding framework of pharmacists’ roles in the emergency department (ED) setting can be presented.

Existing systematic reviews and meta-analyses have explored the role of ED pharmacists broadly, including across various emergency presentations such as sepsis, stroke, and anaphylaxis [6,14]. A previous review by Currie et al. assessed pharmacist interventions during emergency response events but did not isolate outcomes in cardiac arrest or trauma scenarios [14]. In contrast, our paper focuses specifically on cardiac and trauma resuscitations to provide a clearer and more targeted analysis of medication-related and patient-centered outcomes. These two resuscitation scenarios—cardiac arrest and trauma—were selected because they are among the most high-stakes and protocolized emergencies in the ED, and existing reviews have not yet isolated these contexts to determine the pharmacist’s true impact [14]. Including a broader range of conditions (e.g., sepsis or stroke) would introduce clinical heterogeneity and risk diluting the findings. By narrowing our focus, we aim to provide a clearer, evidence-based framework for the integration of clinical pharmacists into resuscitation teams. Our primary objective is to assess the impact of pharmacist involvement on medication processes (e.g., medication errors and time to administration) and patient outcomes (e.g., mortality and morbidity) during cardiac and trauma resuscitations. We also aim to explore perceived barriers and facilitators to pharmacists’ participation in these settings, as described in the existing literature. This focused approach will help inform ED programs and policy-makers seeking to optimize resuscitation team structures for improved patient safety and outcomes.

## 2. Materials and Methods

This scoping review was conducted in accordance with the PRISMA (Preferred Reporting Items for Systematic reviews and Meta-Analyses) guidelines [16]. It was not registered on PROSPERO as the platform typically does not accept scoping reviews. Additionally, given that this review was conducted as part of a capstone project, public registration was not considered necessary. Two investigators (HP, MW) utilized four databases (Medline, Embase, Google Scholar, and Web of Science) to screen for records. To balance thoroughness with feasibility, these databases were chosen because MEDLINE content is included in PubMed and therefore, a separate PubMed search was considered unnecessary. These databases were accessed in September 2024. This was based on a predetermined inclusion and exclusion criteria (Table 1) that took into consideration the inherent broadness of the topic; thus, only isolating results relevant to pharmacists participating in specific cardiac and trauma resuscitations in the ED. Our clinical question is summarized in Table 2 and Table 3. The raw data can be found in the Supplementary Material. The following Medical Subject Heading (MeSH) terms were used for the literature search within the databases: clinical pharmacists, hospital pharmacists, advanced cardiac life support, emergency department, trauma center, medication errors, cardiac arrests, trauma resuscitation, and cardiac resuscitation (Table A1, Table A2 and Table A3). Subsequently, the search results were imported from the databases into the Covidence tool (*n* = 640) and duplicates (*n* = 21) were filtered out. The remaining results (*n* = 619) were subject to an independent abstract and title screening.

No date restrictions were applied to the search as we aimed to capture the evolution of emergency medicine pharmacy practice. Older studies were included to reflect how pharmacists’ roles in resuscitation teams have developed over time and to provide historical context for current practices. While some methods may have changed, these studies remain relevant to understanding the trajectory and impact of pharmacist involvement.

Titles and abstracts were screened independently by two reviewers (HP, MW), with a third reviewer (AL or AT) resolving any disagreements and discrepancies. Two reviewers (HP, MW) independently conducted data extraction using a standardized data collection form. Data extraction was guided by recommendations from the Joanna Briggs Institute (JBI) for scoping reviews [17]. A custom data extraction spreadsheet was developed to capture study characteristics, pharmacist roles, and key clinical outcomes. This form was based on JBI principles.

The reviewers captured key study design details, pharmacist background/training and interventions, and key outcomes such as compliance with ACLS guidelines, reduction in medication errors, survival rates, efficiency in care delivery, and post-resuscitation interventions. Outcomes were categorized a priori as primary (e.g., clinical endpoints such as medication errors, adherence to ACLS protocols, patient morbidity/mortality, and hospital length of stay) and secondary (e.g., feasibility, provider experiences, training requirements, and cost-related outcomes) to guide data extraction and synthesis across heterogeneous studies. A scoping review with narrative synthesis was conducted to highlight the published evidence and clinical outcomes of pharmacist involvement in cardiac and trauma resuscitations. The included article’s risk of bias was evaluated using the Newcastle–Ottawa Scale (NOS) (Table 4) independently by two reviewers (HP, MW) for comparative observational studies to be able to comment on the reliability of the reported data, methodology, and the certainty of the conclusions [18]. The NOS is a validated and pragmatic tool suited to assess bias in observational studies reporting clinical outcomes such as medication errors, mortality, and adherence to ACLS protocols. While more complex tools like ROBINS-I were considered, NOS offered a structured and efficient approach appropriate to the scope and feasibility of this review. Risk of bias was assessed only for studies reporting primary clinical outcomes; studies focused solely on secondary or qualitative outcomes (e.g., feasibility, cost savings, and provider perspectives) were excluded as the NOS is not designed for non-quantitative data.

**Table 1:** Inclusion and exclusion criteria.

Inclusion Criteria	Exclusion Criteria
Pharmacists Cardiac arrest events or events that require emergency resuscitation (including cardiac arrest and major trauma resuscitations) EDs and/or trauma care settings worldwide Randomized and non-randomized control trials (including existing meta-analyses)	ED visits that are not cardiac events or do not require emergency resuscitation (excluding acute stroke, acute hemorrhage, status epilepticus) Unpublished or non-peer-review journals Conference abstracts

**Table 2:** Summary of Primary Outcomes: study design, number of participants, country of origin, type of event, and clinical findings.

Study ID	Design	Sample Size	Country	Event	Primary Outcomes
Amini, 2013 [19]	Retrospective Cohort	100	USA	Trauma	PP decreased overall length of stay in the ED
Bolt, 2015 [7]	Survey	43	CAN	Cardiac	Institutions with PP on the CPR team saw increased ACLS guideline—medication compliance
Bond, 1999 [20]	Statistical Analyses	1029	USA	Cardiac	PP clinical services decreased mortality (40,478 fewer deaths/year)
Cooper, 2007 [21]	Chart Review	46	USA	Cardiac	PP increased ACLS medication compliance (therapy recommendation and drug dosage adjustment)
Currey, 2024 [14]	Systematic Review	30 Full-Texts	AUS	Cardiac Trauma	PP increased compliance with ACLS guidelines and reduced medication errors
Draper, 2008 [8]	Retrospective Analysis	74	USA	Cardiac	PP improved medication compliance and reduced MI- related medication errors—significant PP improved survival to hospital discharge and decreased mortality
Ernst, 2012 [22]	Cross- Sectional Cohort Study	694	USA	Cardiac Trauma	PP reduced medication errors
Fairbanks, 2008 [23]	Quality Improvement	72	USA	ED	PP reduced rate of medication errors
Felh, 2017 [24]	Retrospective Quasi- Experimental	Pre: 234 Post: 157	USA	Cardiac	PP did not cause significant difference in time to hospital discharge
Groth, 2016 [25]	Evaluation of Pilot Program Study	32	USA	Cardiac	PP increased ACLS medication compliance (therapy addition) PP decreased patient escalation of care
Hashemipour, 2013 [26]	Quality Improvement	Pre: 54 Post: 32	USA	Cardiac	PP increased ACLS medication compliance (not significant) PP decreased number of cardiac arrests
Heavner, 2018 [27]	Retrospective Chart Review	Pre: 26 CA Post: 54 CA	USA	Cardiac	PP increased ACLS compliance—significant PP improved acute event survival to hospital admission—significant
Lada, 2007 [28]	Quality Improvement	34	USA	N/A	PP prevented medication errors
Lamkin, 2019 [29]	Retrospective: Cohort Chart Review	1082 Study PP: 782 Control PA: 300	USA	Trauma	PP increased proper use of Lidocaine and decreased medication errors—significant
McAllister, 2017 [30]	Retrospective Analysis	PP: 20 PA: 45	USA	Cardiac	PP increased ACLS medication compliance—significant PP increased survival to hospital admission and discharge—significant
McGinnis, 2022 [13]	Quality Improvement	19-RRT Activations 104 Interventions	USA	Cardiac	PP decreased medication errors
Ray, 2024 [31]	Survey	184	USA	Cardiac	PP optimized preparation, administration of resuscitative medication
Robb, 2003 [32]	Questionnaire	164	UK	Cardiac	PP optimized medication preparation and simplified ACLS algorithm
Roman 2024 [15]	Unblinded Randomized Control Trial	Control: 37 Study: 43	AUS	Trauma	PP reduced medication errors (able to prescribe medications) PP did not cause significant difference in length of stay in ED or hospital

**Table 3:** Summary of Secondary Outcomes: country of origin, type of event, and clinical findings.

Study ID	Country	Event	Secondary Outcomes
Al Harbi, 2006 [33]	USA	Cardiac	Majority of pharmacists were BLS-certified (*n* = 30) Fewer pharmacists were ACLS-certified (*n* = 20) ACLS-trained pharmacists (*n* = 16/20) were confident in providing drug information in a CPR situation vs. non-ACLS-trained pharmacists (*n* = 4/27) ACLS-trained pharmacists (*n* = 14/20) were confident in recommending a drug during a CPR situation vs. non-ACLS-trained pharmacists (*n* = 4/27)
Bolt, 2015 [7]	CAN	Cardiac	Majority (*n* = 30/43) of organizations had less than 25 FTE pharmacists Majority (*n* = 27/43) of organizations had less than 50% of pharmacists who completed residency Several (*n* = 8/43) organizations cited staff coverage issues as barriers to further pharmacist integration on code teams
Bond, 1999 [20]	USA	Cardiac	Pharmacists’ services in 4 key areas were associated with cost avoidance through mortality prevention Clinical research services Drug information Drug histories CPR team participation
Cooper, 2007 [21]	USA	Cardiac	There were 17 FTE pharmacists and 4 clinical specialist FTEs All pharmacists were either certified in ACLS or passed a certification test to participate oin CPR teams
Ernst, 2012 [22]	USA	Cardiac Trauma	Clinical pharmacists are available 10 h/day on a non- on-call basis, allowing for comparison of PA vs. PP
Felh, 2017 [24]	USA	Cardiac	20 pharmacists comprised of staff pharmacists, pharmacy residents
Groth, 2016 [25]	USA	Cardiac	Program was developed by 2 ACLS pharmacists trained in clinical care and emergency medicine
Hale, 2009 [34]	USA	Trauma	27/161 trauma centers (17%) that did not consider integration of pharmacists into trauma team cited staffing difficulties 19/161 trauma centers (12%) that did not consider integration of pharmacists into trauma team cited cost concerns
Hashemipour, 2013 [26]	USA	Cardiac	75.6% of institution pharmacists were in practice for 5+ years—ACLS- and BLS-certified Pharmacists reported increased confidence following training program completion Pharmacists are rotated to cover for code pharmacists’ absence
Heavner, 2018 [27]	USA	Cardiac	Lead pharmacist, PGY2 pharmacy residents acquired ACLS training
Lada, 2007 [28]	USA	N/A	The extrapolated cost avoidance for a one-year period = USD $3,089,328.
Machado, 2003 [35]	USA	Cardiac	Most participating institutions (*n* = 90/149) had 5 FTE pharmacists Most non-participating institutions (*n* = 191/286) had 5 FTE pharmacists Majority of pharmacists (*n* = 301/381) pharmacists were CPR-trained Fewer participant pharmacists (*n* = 140/380) were ACLS-trained Fewer non-participant pharmacists (*n* = 118/723) were ACLS-trained 78% of routine CPR participant emergency pharmacists felt they were adequately trained to respond to codes (*p* < 0.001) 25.4% of non-participants felt adequately trained (*p* < 0.001)
Marlowe, 2005 [36]	USA	Cardiac	Pharmacists reported increased confidence on a scale (0–5) in regards to various code team responsibilities (pre- vs. post-program) Calculate doses of resuscitation medications to be drawn up 3.54 ± 0.43 vs. 3.96 ± 0.2; *p* = 0.015
McAllister, 2017 [30]	USA	Cardiac	Annual cost avoidance provided by a single full-time pharmacist = USD$ 321,308 per year
Ray, 2024 [31]	USA	Cardiac	Most participants (38%) had 4–7 years of practical experience Majority of participants (61%) reported 19–24 h of dedicated EMP coverage
Robb, 2003 [32]	UK	Cardiac	All pharmacists who attended cardiac arrest events received 4H training in BLS and 8H training in ACLS Resident pharmacists attended arrests overnight, on weekends; enabled 24H coverage
Roman 2024 [15]	AUS	Trauma	EM Pharmacists have at least 2 years of clinical experience in hospital pharmacy practice EM pharmacists responded to trauma callouts from Monday to Friday (07:00–21:00 h)
Shimp, 1995 [37]	USA	Cardiac	BLS = 66/160 pharmacy graduates who responded (41%) On a 5-point scale, all pharmacists ranked their confidence as 3.2 ± 1.2 in their ability to perform CPR-BLS Community pharmacists ranked as 3.0 ± 1.1 Hospital pharmacists ranked as 3.3 ± 1.2
Toma, 2007 [38]	USA Puerto Rico	Cardiac	Majority of staff pharmacists were BLS-trained (*n* = 62) but fewer were ACLS-trained (*n* = 46) Majority of clinical pharmacists were BLS-trained (*n* = 67) and a similar number were ACLS-trained (*n* = 55) Majority of PGY1 pharmacists were BLS-trained (*n* = 91) but fewer were ACLS-trained (*n* = 77)—highest proportion Majority of staff pharmacists were BLS-trained (*n* = 48) and a similar number were ACLS-trained (*n* = 41)
Wanbon, 2015 [39]	CAN	N/A	Many pharmacists (*n* = 27/56) had completed hospital residency Majority of pharmacists (*n* = 30/52) were CPR-certified but a lesser amount (*n* = 17/52) were ACLS- certified

**Table 4:** Newcastle–Ottawa risk of bias assessment [18].

	Selection				Comparability	Outcome		
Reference	D1	D2	D3	D4	D5	D6	D7	D8
Amini, 2013 [19]	** 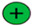 **	** 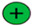 **	** 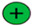 **	** 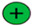 **	** 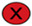 **	** 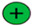 **	** 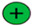 **	** 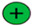 **
Bolt, 2015 [7]	** 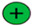 **	** 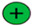 **	** 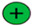 **	** 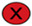 **	** 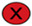 **	** 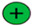 **	** 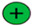 **	** 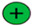 **
Bond, 1999 [20]	** 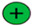 **	** 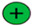 **	** 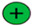 **	** 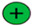 **	** 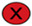 **	** 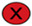 **	** 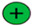 **	** 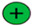 **
Cooper, 2007 [21]	** 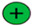 **	** 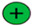 **	** 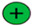 **	** 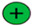 **	** 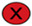 **	** 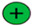 **	** 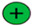 **	** 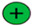 **
Currey, 2024 [14]	** 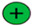 **	** 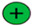 **	** 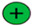 **	** 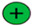 **	** 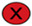 **	** 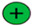 **	** 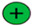 **	** 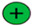 **
Draper, 2008 [8]	** 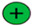 **	** 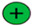 **	** 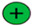 **	** 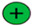 **	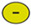	** 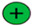 **	** 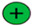 **	** 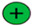 **
Ernst, 2012 [22]	** 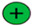 **	** 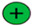 **	** 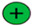 **	** 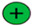 **	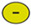	** 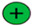 **	** 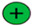 **	** 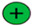 **
Fairbanks, 2008 [23]	** 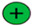 **	** 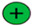 **	** 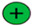 **	** 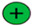 **	** 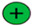 **	** 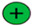 **	** 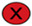 **	** 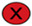 **
Feih, 2017 [24]	** 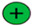 **	** 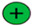 **	** 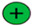 **	** 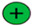 **	** 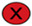 **	** 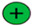 **	** 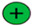 **	** 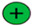 **
Groth, 2016 [25]	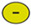	** 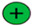 **	** 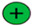 **	** 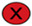 **	** 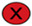 **	** 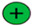 **	** 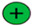 **	** 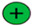 **
Heavner, 2018 [27]	** 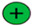 **	** 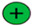 **	** 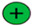 **	** 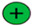 **	** 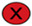 **	** 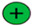 **	** 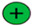 **	** 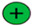 **
Lada, 2007 [28]	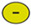	** 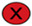 **	** 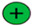 **	** 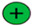 **	** 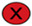 **	** 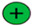 **	** 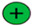 **	** 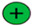 **
Lamkin, 2019 [29]	** 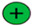 **	** 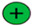 **	** 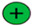 **	** 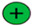 **	** 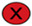 **	** 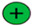 **	** 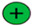 **	** 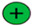 **
McAllister, 2017 [30]	** 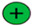 **	** 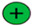 **	** 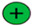 **	** 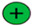 **	** 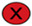 **	** 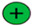 **	** 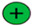 **	** 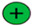 **
McGinnis, 2022 [13]	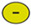	** 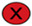 **	** 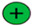 **	** 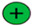 **	** 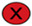 **	** 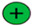 **	** 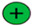 **	** 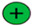 **
Ray, 2024 [31]	** 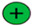 **	** 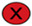 **	** 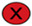 **	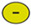	** 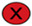 **	** 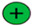 **	** 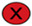 **	** 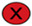 **
Robb, 2003 [32]	** 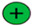 **	** 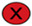 **	** 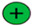 **	** 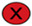 **	** 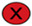 **	** 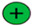 **	** 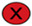 **	** 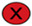 **
Roman, 2024 [15]	** 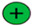 **	** 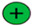 **	** 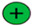 **	** 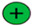 **	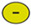	** 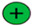 **	** 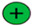 **	** 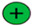 **
Wanbon, 2015 [39]	** 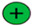 **	** 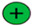 **	** 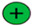 **	** 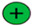 **	** 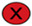 **	** 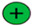 **	** 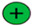 **	** 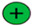 **
Judgement (a) High: 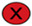 (b) Unclear: 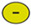 (c) Low: 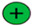 D1: Was the exposed group representative? (Selection bias.) D2: How was the non-exposed group selected? (Selection bias.) D3: How was the exposure ascertained? (Selection bias.) D4: Did the study demonstrate that the outcome of interest was not present at the start of the study? (Selection bias.) D5: Comparability of groups on the basis of the design or analysis controlled for confounders (Comparability bias.) D6: How was the outcome assessed? (Outcome bias.)D7: Was follow-up long enough for outcomes to occur? (Outcome bias.)D8: Was the follow-up of subjects adequate? (Outcome bias.)								

## 3. Results

### 3.1. Study Selection

**Figure 1:**
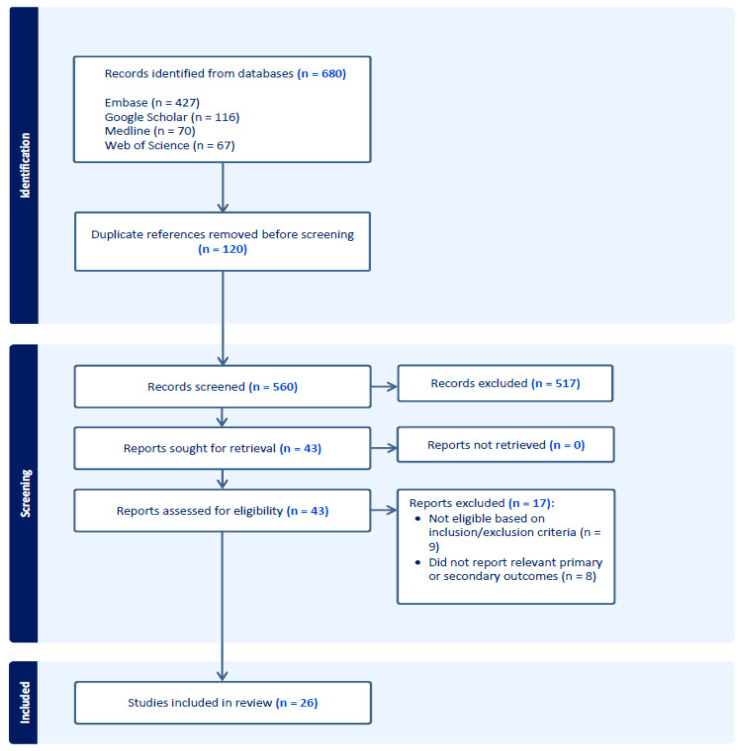
PRISMA flow diagram of study selection.

The literature search identified 680 studies, of which 120 duplicates were removed. Following the title and abstract screening, 43 studies were selected for full-text review according to the inclusion and exclusion criteria. A total of 26 studies were included in this review. Studies were excluded for the following reasons: (1) study outcomes did not match our primary/secondary outcomes, or (2) type of study did not meet the criteria. Refer to Figure 1.

### 3.2. Study Characteristics

The information extracted to describe the research articles included the year, study design, country, and type of study (cardiac or trauma resuscitation). The majority of studies were published in 2010 or onwards, while nine were in the 2000s and two in the late 1990s. Although the literature search was global, most of the studies were conducted in the USA, Canada, and Australia. Among these, the included studies consisted of six retrospective cohort studies, nine surveys, four quality improvement studies, one cross-sectional cohort study, one questionnaire-based study, one pilot study, one statistical analysis, one unblinded randomized controlled trial, and one systematic review. More than half of the studies were specific to cardiac resuscitations, while the others focused on trauma resuscitations. All the studies were conducted in a hospital setting, specifically at level-one trauma centers with highly specialized pharmacy services in the EDs. The study sample sizes ranged from 28 to 1082 participants amongst the various articles.

### 3.3. Outcomes

The findings of this scoping review were extracted from 26 studies. Within primary outcomes, most studies reported compliance with ACLS guidelines (*n* = 7) and medication errors (*n* = 8) comparing the effects of pharmacists’ presence (PP) with their absence (PA). These surrogate markers are important because adherence to ACLS protocols and medication accuracy are directly linked to timely and appropriate resuscitation efforts, which are known to influence patient survival and recovery. However, it is important to recognize that while these outcomes indicate improved process measures, they do not fully capture the complexity of patient-centered clinical endpoints. Fewer studies reported mortality (*n* = 3), morbidity (*n* = 1), survival to hospital admission/discharge (*n* = 3), and length of stay in the ED/hospital (*n* = 2), which are classified as direct patient outcomes. Multiple studies reported pharmacist characteristics as secondary outcomes including level of certification (*n* = 8) and level of confidence concerning ACLS (*n* = 6). Other secondary outcomes included logistical considerations such as staffing (*n* = 7) and cost avoidance associated with the pharmacists’ presence (*n* = 4).

Pharmacists’ contributions were most notable in cardiac resuscitation (*n* = 12) compared to trauma (*n* = 2) with respect to ACLS compliance and medication errors. Three studies demonstrated that compliance with ACLS guidelines was significantly increased with the presence of a pharmacist compared to absence of a pharmacist. Optimization of medication preparation and administration emerged as a core benefit, with an increased number of correct medications administered as a result [31,32]. The remaining studies consistently observed a positive trend in the latter, although statistical significance was not reported. Procedures such as time to medication administration (e.g., analgesia and sedation) and emergency resuscitation through pharmacologic measures were other aspects where pharmacists’ involvement improved adherence to ACLS protocols [29,32]. Several studies noted that the overall number of medication errors also decreased when pharmacists were present on the code team [13,23,28,29]. This reduction in medication errors is critical because errors during resuscitations can lead to adverse drug events, compromising patient safety and clinical outcomes.

Although limited studies reported direct patient care outcomes, they suggested positive trends towards a decrease in morbidity and mortality with the presence of a pharmacist. Evidence of improved survival was less robust in trauma resuscitations; however, studies highlighted pharmacists’ ability to expedite pre-trauma and post-resuscitative care [15,29,31].

A commonly reported surrogate endpoint was pain, which was characterized to impair respiratory function, immune response, wound healing, and worsen patient outcomes by eliciting metabolic stress response in patients with severe trauma. Appropriate early pain control in trauma patients facilitated by pharmacists was associated with improved patient outcomes such as reduced stress response, a shorter hospital length of stay, early wound healing, reduced post-traumatic chronic pain, and improvement in quality of life [15,40,41]. Our review identified that median time to analgesia administration during trauma significantly decreased by approximately 8 to 23 min with the presence of a pharmacist [15,19,29]. Effective trauma management may enhance long-term patient outcomes (i.e., mortality and morbidity) while minimizing the need for more intensive treatment methods and escalation to higher levels of critical care [22].

Several studies observed substantial cost reductions in the range of USD 300,000 to USD 1,000,000 annually [28,30]. By providing drug therapy consultation and improving medication accuracy through therapy recommendations, pharmacists minimized the need for escalated care, additional treatments, and reduced prolonged hospital stays [28,30]. These economic benefits further reinforce the value of pharmacist integration as a cost-effective intervention that supports both clinical and operational healthcare goals. Inadequate staffing coverage by certified pharmacists was cited as a barrier to increased pharmacist participation on code teams [34]. This shortage largely stems from institutional policies that do not mandate ACLS certification for pharmacists working in the emergency department [37]. Our review identified notable variability in the training and certification levels among emergency department pharmacists: while most pharmacists held basic Cardiopulmonary Resuscitation (CPR) and Basic Life Support (BLS) certifications, a substantial proportion lacked formal ACLS accreditation. Pharmacy residents were more likely to possess ACLS certification, highlighting a gap between training stages. Several healthcare system factors contributed to this gap, including the absence of standardized ACLS certification programs tailored for pharmacists and limited funding support. Moreover, pharmacists faced barriers such as scarce opportunities to apply ACLS skills in practice, which, combined with insufficient staffing levels, discouraged ongoing certification or recertification efforts in both BLS and ACLS training [20,34,37]. The lack of ACLS certification has a direct impact on pharmacists’ confidence and competence in emergency code situations. Studies show that ACLS-certified pharmacists reported significantly higher confidence levels, enabling them to safely and effectively fulfill critical responsibilities such as medication administration and dose-adjustment recommendations during resuscitations [26,33,36]. Without appropriate certification and training, pharmacists’ ability to fully integrate into emergency teams and contribute to improved patient care may be compromised.

## 4. Discussion

Although the multidimensional role of pharmacists has recently gained recognition in the hospital setting, there are still multiple domains that can benefit from their expertise. The results from this narrative review suggest that pharmacists’ involvement in cardiac and trauma resuscitations is associated with positive patient outcomes. Specifically, our findings align with the prior literature reporting reduced medication errors and improved ACLS guideline adherence when pharmacists participate in emergency responses. The participation of pharmacists was associated with improved and expedited trauma care, specifically the time to analgesia. ACLS accreditation increased pharmacists’ confidence to perform ACLS-associated tasks in rapid-response teams. Although staffing shortages were identified as a principal barrier to including pharmacists on the team, financial evaluations revealed that their participation substantially reduced healthcare costs.

Staffing shortages and inconsistent ACLS certification are principal barriers to fully integrating pharmacists into emergency resuscitation teams. These issues limit their availability and may undermine confidence and competence during critical events. The absence of mandatory ACLS certification and limited institutional support for standardized training reflect systemic gaps that need addressing. Additionally, low staff coverage and few opportunities to apply ACLS skills reduce motivation to pursue or maintain certification, perpetuating underutilization. This limits pharmacists’ potential to improve patient outcomes. Yet, financial analyses show their involvement reduces healthcare costs, highlighting the value of investment. Addressing these barriers through policy changes, funding, and supportive environments can empower pharmacists to improve ACLS adherence and reduce medication errors [7,8,13,14,25,27,28].

To our knowledge, this is the first scoping review to specifically assess the impact of pharmacists on patient outcomes within cardiac and trauma resuscitations, distinguishing it from broader reviews of in-hospital resuscitation or rapid response events. Our review focuses on cardiac and trauma resuscitations to allow for a more granular understanding of pharmacist contributions during the most time-critical and pharmacologically demanding emergencies. Multiple studies demonstrated that the presence of pharmacists improved adherence to ACLS guidelines. This was achieved through optimized medication preparation and administration, time reduction to administer life-saving medications, as well as providing appropriate pharmacotherapy consults to the healthcare team.

Consistent with the previous literature, our synthesized data suggested that pharmacists’ involvement is correlated to a reduction in medication errors. This correlation reinforces the role of pharmacists as medication safety experts, crucial in emergency settings where the risk for errors is high due to urgency and complexity. As such, the integration of pharmacists into resuscitation teams offers a pathway to address existing gaps in emergency and trauma care practices. Healthcare institutions can utilize pharmacists’ expertise in pharmacotherapy to support appropriate and efficient medication practices in ACLS, thereby leading to improved patient outcomes. Our study revealed the need for healthcare institutions to standardize training and certification programs for pharmacists. As confidence in participating in code response teams directly improved with ACLS training, this certification should be implemented as a recommended requirement for pharmacists employed in the ED. Healthcare institutions should provide opportunities to support pharmacists in receiving ACLS training. The integration of a pharmacist can result in yearly cost savings of up to USD 1,000,000. Administrators should recognize that investing in pharmacist training may not only yield significant long-term economic benefits but also improve the quality of patient care.

Furthermore, there is a lack of evidence concerning pharmacists’ impact on patient outcomes as few studies reported direct clinical outcomes such as mortality, morbidity, survival to hospital admission/discharge, and length of stay. Interpretation of mortality outcomes is limited by the small number of studies reporting these data, heterogeneous patient populations, low-quality study methodologies, and potential confounding factors that were not consistently controlled for, which restricts the ability to draw definitive conclusions about the direct impact of pharmacist involvement on survival rates. Despite this, we identified that pharmacists’ participation leads to a reduction in the time to administration of analgesia in trauma settings. It has been well established that prolonged acute pain is linked to worsening long-term patient outcomes [15,40,41]. The addition of pharmacists to trauma teams may be a strategic avenue to facilitate effective pain management, which may lead to reduced hospital length of stay and better quality of life. More quantitative research is warranted to investigate whether pharmacists can improve long-term health outcomes.

As previously mentioned, the existing meta-analysis by Currie et al. captured the broader role of clinical pharmacists during in-hospital resuscitation or medical response events [14]. While our findings were similar to theirs, our study differed by focusing specifically on the two critical care settings including cardiac and trauma resuscitations. While Currie et al. broadly described pharmacist roles during in-hospital code events, our review provides a more targeted synthesis of pharmacist contributions during cardiac and trauma-specific resuscitations, which are arguably the most time-sensitive and pharmacologically intense situations.

Unlike their review, which aggregated diverse response scenarios, we identified domain-specific outcomes such as time to analgesia in trauma and ACLS adherence in cardiac events. Unlike earlier reviews that have grouped pharmacist involvement within general code blue or rapid response teams, our scoping review disaggregates data to focus on high-stakes resuscitations, namely cardiac arrest and trauma, where medication decisions are both an emergency and impactful. This distinction enables a more refined understanding of pharmacist contributions to life-saving care.

The dynamic and evolving scope of pharmacy practice in the ED is not always fully known and understood by all members of the healthcare team, including pharmacists themselves. This review highlights a need for a perspective shift in pharmacists’ scope of practice. Based on these findings, it appears that pharmacists contribute meaningfully to life-saving interventions in both clinical and technical aspects of resuscitation. However, overcoming resistance to change in the healthcare industry may be challenging. Advocating for future growth in the profession should be a priority for pharmacy associations.

There are several limitations to this scoping review. The majority of studies were conducted in North America, predominantly in the United States and Canada. This limits generalizability as findings may not be directly applicable to countries with different healthcare systems. The predominance of U.S. and Canadian studies highlights a gap in understanding how pharmacists function in emergency resuscitation roles internationally. Additionally, many studies were performed in level-one trauma centers with highly specialized pharmacy services, which may not reflect conditions in smaller community or private hospitals. A high level of heterogeneity was observed in study design and outcome reporting. As outcomes were inconsistently measured across studies, it was challenging to evaluate an effect measure. Many of the studies had small sample sizes and missing statistics (e.g., *p*-values), which limited our ability to conduct any meaningful statistical analyses or meta-analyses. Finally, there is a lack of mortality and morbidity data reported from the studies, which is a key barrier to assessing the impact of pharmacists’ involvement in direct patient outcomes.

Our research demonstrates that while significant advancements have been made in the scope of practice for ED pharmacists, there are still many opportunities to fully leverage and integrate pharmacists’ expertise into emergency care. Although several studies have demonstrated an association between improved hospital efficiency and the integration of pharmacists into care teams, there remains a lack of research exploring the patient journey throughout their hospital stay, as well as limited evidence on impacts related to mortality and morbidity. Given the consistency of findings around medication safety and operational efficiency, our review supports the broader integration of pharmacists into resuscitation teams and underscores the importance of policy-level change to strongly encourage ACLS training and full participation in code response protocols.

Ideally, future research should aim for rigorous designs such as multicenter randomized controlled trials assessing direct impacts on mortality and morbidity to provide high-level evidence of pharmacists’ roles in resuscitation. However, recognizing the challenges in conducting such trials, pragmatic approaches including observational cohort studies using existing clinical data and mixed-methods research exploring team dynamics and process outcomes are important intermediate steps.

Research questions could target measurable outcomes like medication errors and time to medication administration, while also exploring impacts on patient survival where feasible. For future investigations, we propose a standardized core outcome set with practical, validated metrics to enable consistent reporting and reduce heterogeneity. This approach will enable consistent and comparable data reporting across healthcare institutions, reducing inter-study heterogeneity. Achieving reproducibility in outcomes will provide a clearer and more reliable understanding of pharmacists’ impact on cardiac and trauma resuscitations, laying the groundwork for more robust evidence in this area.

## 5. Conclusions

While our findings are limited by high heterogeneity and variable study quality among the included literature, available evidence suggests positive trends highlighting the potential clinical benefits of pharmacists’ involvement in cardiac and trauma resuscitations. These benefits include potential improvements in indirect patient outcomes, such as improved adherence to ACLS guidelines, reduced medication errors, expedited medication administration, and significant cost savings. However, evidence supporting direct clinical outcomes like mortality, morbidity, survival, and length of stay remains limited and inconclusive due to heterogeneity, study design limitations, and confounding factors. These trends underscore the need for further investigation. Future studies should prioritize developing a standardized core outcome set with clear definitions to minimize inter-study variability and focus on capturing direct, meaningful impacts on patient health outcomes to strengthen the evidence base.

## Data Availability

All data generated or analyzed are included in this published article.

## Supplementary Materials

The following supporting information can be downloaded at: https://www.mdpi.com/article/10.3390/pharmacy13040089/s1, Table S1: The raw data [7,8,13,14,15,19,20,21,22,23,24,25,26,27,28,29,30,31,32,33,34,35,36,37,38,39].

## Abbreviations

The following abbreviations are used in this manuscript:

ACLS	Advanced cardiac life support
AUS	Australia
BLS	Basic life support
CA	Cardiac arrest
CAN	Canada
CPR	Cardiopulmonary resuscitation
ED	Emergency department
EM	Emergency medicine
EMP	Emergency medicine pharmacists
MI	Myocardial infarction
PA	Pharmacist absent
PGY1	Post-graduate year 1
PGY2	Post-graduate year 2
PP	Pharmacist present
RRT	Rapid response team
USA	United States of America
UK	United Kingdom

## Appendix A

**Table A1:** Search strategy used for Embase database.

#	Query	Results from September 2024
1	(clinical pharmacist or pharmacist).mp. [mp = title, book title, abstract, original title, name of substance word, subject heading word, floating sub-heading word, keyword heading word, organism supplementary concept word, protocol supplementary concept word, rare disease supplementary concept word, unique identifier, synonyms, population supplementary concept word, anatomy supplementary concept word]	105, 162
2	exp Heart Arrest/	134, 089
3	exp cardiopulmonary resuscitation/or exp advanced cardiac life support/	135, 798
4	exp resuscitation/	141, 227
5	(cardiac resuscitation or trauma resuscitation or cardiac arrest* or advanced cardiac life support or ACLS or CPR or code blue or trauma response team).mp. [mp = title, abstract, heading word, drug trade name, original title, device manufacturer, drug manufacturer, device trade name, keyword heading word, floating subheading word, candidate term word]	101, 636
6	exp advanced cardiac life support/	836
7	exp emergency ward/	234, 541
8	exp emergency health service/	354, 546
9	exp emergency medicine/	49, 973
10	(emergency department or emergency ward or emergency medicine or trauma cent*).mp. [mp = title, abstract, heading word, drug trade name, original title, device manufacturer, drug manufacturer, device trade name, keyword heading word, floating subheading word, candidate term word]	365, 707
11	1 or 2	134, 089
12	3 or 4 or 5 or 6	254, 491
13	7 or 8 or 9 or 10	449, 850
14	11 and 12 and 13	194
15	11 amd 12	661
16	Limit 15 to Embase	425

**Table A2:** Search strategy used for Medline database.

#	Query	Results from September 2024
1	((clinical pharmacist or pharmacist).mp. [mp = title, book title, abstract, original title, name of substance word, subject heading word, floating sub-heading word, keyword heading word, organism supplementary concept word, protocol supplementary concept word, rare disease supplementary concept word, unique identifier, synonyms, population supplementary concept word, anatomy supplementary concept word]	23, 024
2	exp Heart Arrest/	58, 590
3	exp cardiopulmonary resuscitation/or exp advanced cardiac life support/	23, 543
4	(cardiac arrest* or trauma resuscitation or cardiac resuscitation or ACLS or CPR or code blue or trauma response team).mp. [mp = title, book title, abstract, original title, name of substance word, subject heading word, floating sub-heading word, keyword heading word, organism supplementary concept word, protocol supplementary concept word, rare disease supplementary concept word, unique identifier, synonyms, population supplementary concept word, anatomy supplementary concept word]	61, 585
5	exp emergency service, hospital/or exp trauma centers/	104, 613
6	(emergency medicine or emergency department or emergency ward or trauma cent*).mp. [mp = title, book title, abstract, original title, name of substance word, subject heading word, floating sub-heading word, keyword heading word, organism supplementary concept word, protocol supplementary concept word, rare disease supplementary concept word, unique identifier, synonyms, population supplementary concept word, anatomy supplementary concept word]	176, 604
7	2 and 5	1, 179
8	exp Pharmacists/	23, 136
9	1 or 8	35, 346
10	2 or 3 or 4	96, 620
11	5 or 6	211, 697
12	9 and 10	70
13	9 and 10 and 11	25

**Table A3:** Search strategy used for Google Scholar database.

#	Query	Results from September 2024
1	(“clinical pharmacists” OR “hospital pharmacists”) AND (“emergency department”) AND (“cardiac arrest” OR “trauma resuscitation”) AND (“advanced cardiac life support” OR “ACLS”)	116
